# Identification and Characterization of a Fatty Acid- and Retinoid-Binding Protein Gene (*Ar-far-1*) from the Chrysanthemum Foliar Nematode, *Aphelenchoides ritzemabosi*

**DOI:** 10.3390/ijms20225566

**Published:** 2019-11-07

**Authors:** Shan-Wen Ding, Dong-Wei Wang, Yu Xiang, Chun-Ling Xu, Hui Xie

**Affiliations:** 1 Laboratory of Plant Nematology and Research Center of Nematodes of Plant Quarantine, Department of Plant Pathology/Guangdong Province Key Laboratory of Microbial Signals and Disease Control, College of Agriculture, South China Agricultural University, Guangzhou 510642, China; dingshanwen@foxmail.com (S.-W.D.); www.wdw@163.com (D.-W.W.); xiangyu@agri.gov.cn (Y.X.); xuchunling@scau.edu.cn (C.-L.X.); 2 Hunan Plant Protection Institute, Hunan Academy of Agricultural Science, Changsha 410125, China

**Keywords:** *Aphelenchoides ritzemabosi*, fatty acid- and retinoid-binding protein, expression pattern, RNAi, reproduction, pathogenicity

## Abstract

The chrysanthemum foliar nematode (CFN), *Aphelenchoides ritzemabosi*, is a migratory, plant-parasitic nematode that is widely distributed and infects the aboveground parts of many plants. The fatty acid- and retinoid-binding proteins (FAR) are nematode-specific proteins that are involved in the development, reproduction, and infection of nematodes and are secreted into the tissues to disrupt the plant defense reaction. In this study, we obtained the full-length sequence of the FAR gene (*Ar-far-1*) from CFN, which is 727 bp and includes a 546 bp ORF that encodes 181 amino acids. *Ar*-FAR-1 from CFN has the highest sequence similarity to *Ab*-FAR-1 from *A. besseyi*, and they are located within the same branch of the phylogenetic tree. Fluorescence-based ligand-binding analysis confirmed that recombinant *Ar*-FAR-1 was bound to fatty acids and retinol. *Ar-far-1* mRNA was expressed in the muscle layer, intestine, female genital system, and egg of CFN, and more highly expressed in females than in males among the four developmental stages of CFN. We demonstrated that the reproduction number and infection capacity of CFN decreased significantly when *Ar-far-1* was effectively silenced by in vitro RNAi. *Ar-far-1* plays an important role in the development, reproduction, infectivity, and pathogenesis of CFN and may be used as an effective target gene for the control of CFN. The results provide meaningful data about the parasitic and pathogenic genes of CFN to study the interaction mechanism between plant-parasitic nematodes and hosts.

## 1. Introduction

The chrysanthemum foliar nematode (CFN), *Aphelenchoides ritzemabosi*, is an obligate ecto- and endoparasite of the aboveground parts of plants [1,2]. CFN has a range of hosts that includes almost 200 different plant species and has been listed as a quarantine plant pest in some countries and regions [3,4].

The molecular biology of plant-parasitic nematodes has attracted much attention from scholars who have done a great deal of research; however, there are few studies on the molecular biology of CFN. To our knowledge, there has as yet been no report on the functional genes of CFN, and the only phylogenetic analyses of foliar nematodes that have been conducted have been based on the small subunit ribosomal DNA of CFN [5,6] and transcriptome analysis of CFN [7].

The fatty acid- and retinoid-binding (FAR) proteins are helix-rich, lipid-binding proteins that are unique to nematodes and may play an important role in acquiring fatty acids and retinoids from the host [8]. After nematodes secrete FAR proteins into the host tissues that they occupy, these FAR proteins may interfere with intercellular lipid signaling to manipulate the defense reactions of the host and could represent potential targets for new nematicides [8,9]. The *Gp*-FAR-1 from *Globodera pallida* has been shown to accumulate on the nematode body surface and is thought to interfere with plant lipoxygenase (LOX)-mediated defense signaling [10]. It has been demonstrated that the FAR protein from the root-knot nematode *Meloidogyne javanica* may adjust expression of lipid-, cell wall-, and phenylpropanoid-related genes during nematode infection of hosts [11], the FAR protein in *M. incognita* possibly influenced the ability of nematodes to move [12], and the *Rs-far-1* from *Radopholus similis* was involved in the regulation of allene oxide synthase (AOS) expression in *Anthurium thaliana* [13].

With the development of transcriptome sequencing technology, the nematode-specific FAR proteins were being extensively discovered and studied [8,9,10,12,13,14,15,16,17,18,19,20,21,22,23,24,25,26,27]. According to our data, at least seventy FAR proteins have been identified in thirty-nine species of nematodes [8,9,10,12,13,14,15,16,17,18,19,20,21,22,23,24,25,26,27]. In plant-parasitic nematodes, twenty-six FAR proteins from seventeen species have been identified, most of which are Tylenchida nematodes, while only two species, *A. besseyi* and *Bursaphelenchus xylophilus,* are Aphelenchida nematodes.

In this study, the *Ar*-FAR-1 protein was obtained through prokaryotic expression after the FAR protein gene (*Ar-far-1*) of CFN was cloned using the transcriptome sequencing data of CFN, and its structure and features were analyzed, and its binding activity was detected. We also studied the localization and expression of *Ar-far-1* in CFN, and the role of *Ar-far-1* in reproduction and pathogenesis of CFN. This is the identification and functional study of the first functional gene in CFN.

## 2. Results

### 2.1. Cloning and Analysis of Full-Length Ar-far-1 from CFN

**Figure 1:**
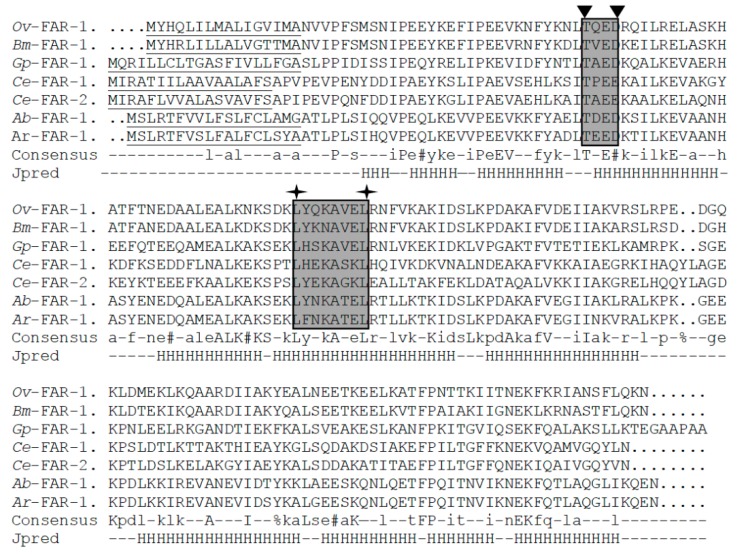
Multiple sequence alignment and structural predictions of the *Aphelenchoides ritzemabosi Ar-*FAR-1 protein and other FAR proteins from selected nematodes. *Ov*-FAR-1: *Onchocerca volvulus* FAR (Q25619); *Bm*-FAR-1: *Brugia malayi* FAR (Q93142); *Gp*-FAR-1: *Globodera pallida* FAR (CAA70477); *Ce*-FAR-1: *Caenorhabditis elegans* FAR-1 (NP_001254978); *Ce*-FAR-2: *Caenorhabditis elegans* FAR-2 (NP_499011); *Ab*-FAR-1: *Aphelenchoides besseyi* FAR-1; *Ar-*FAR-1: *A. ritzemabosi* FAR-1 (KX816796). The putative signal peptide sequences in FAR proteins were underlined. The shaded boxes between two inverted triangles indicate potential casein kinase II (CKII) phosphorylation sites. The shaded boxes between two cross stars indicate potential protein kinase C (PKC) phosphorylation sites. In the consensus line, *uppercase letters* refer to conservation of amino acid position across the entire array, and *lowercase letters* refer to cases where that amino acid occurred in that position in more than half of the sequences. Other symbols are as follows: % for either F or Y, and # for anyone of NDQEBZ. The Jpred line shows the secondary structure prediction from submission of the multiple alignment to the Jpred secondary structure prediction program. H Prediction for α-helix; gaps regions for which no structural prediction emerged. No β-structure was predicted by Jpred or any other secondary structure prediction programs.

**Figure 2:**
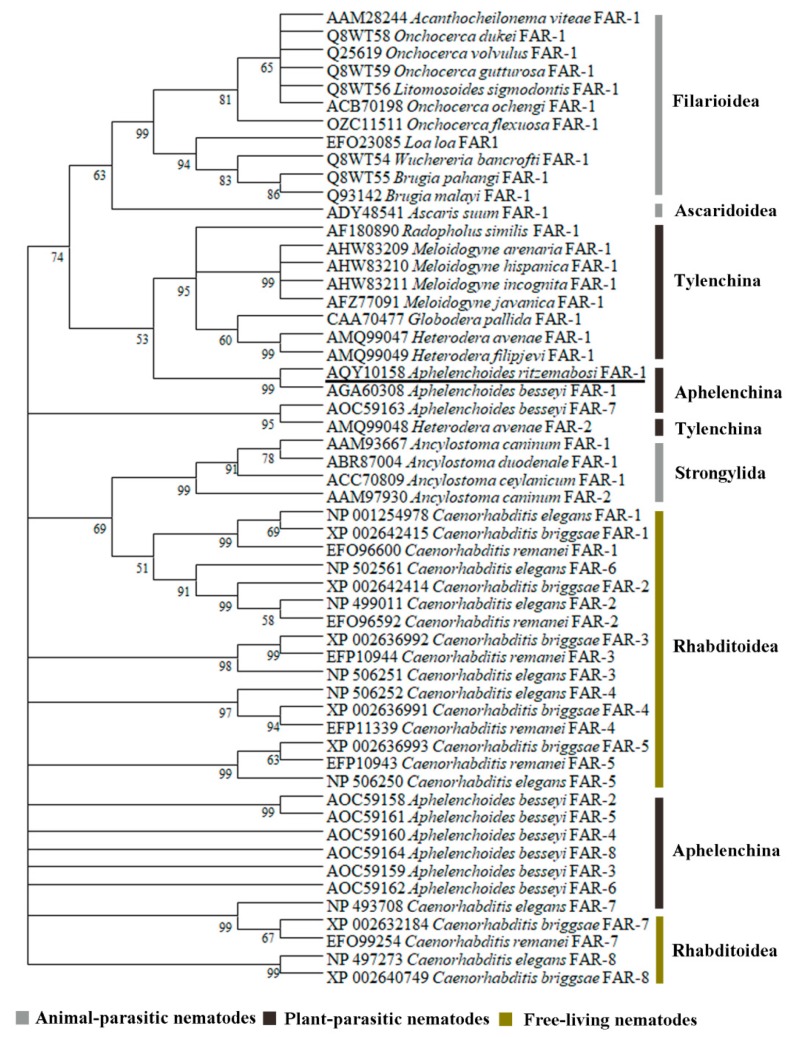
Phylogenetic relationships between *Aphelenchoides ritzemabosi Ar*-FAR-1 protein and FAR proteins from twenty-eight other nematodes. This phylogram was constructed with the amino acid sequences from 55 FAR proteins from 28 different nematodes using neighbor-joining algorithm to describe their evolutionary relationships. The numbers below the branches indicated the bootstrap values, which were calculated from 1000 replicates. *A. ritzemabosi Ar*-FAR-1 was underlined.

A full-length sequence of the FAR gene (*Ar-far-1*) from CFN was amplified using the specific primers (Table 1) and analyzed with software [7]. A full-length cDNA sequence of *Ar-far-1* was 727 bp and included a 546 bp open reading frame (ORF) that encodes 181 amino acids (GenBank accession number KX816796) (Figures S1 and S2). The full-length DNA of *Ar-far-1* was 766 bp and included five exons (100, 230, 60, 106, and 50 bp) and four introns (44, 83, 48, and 45 bp) (Figures S3 and S4). The molecular weight and theoretical isoelectric point of *Ar*-FAR-1 protein were 20.52k Da and 6.22, respectively. There was an 18 amino acid signal peptide with a cleavage site between Ala18 and Glu19 at the *N*-terminus and no *N*-linked glycosylation site. The blastp program analysis showed that *Ar-*FAR-1 is homologous to other FARs from other nematodes, with the highest similarity to *Ab*-FAR-1 from *A. besseyi* (91% identity, *E*-value = 9e–115), and includes a conserved domain similar to that in *Gp*-FAR-1 (Interval = 31–170, *E*-value = 1.94e–44, Pfam ID PF05823). Like the six FAR proteins with known 3–D structures from other nematodes (*A. besseyi*, *Brugia malayi, C. elegans*, *G. pallida*, and *Onchocerca volvulus*), the Ar-FAR-1 protein contain conserved potential casein kinase II phosphorylation sites and protein kinase C phosphorylation sites. Secondary structure prediction algorithms applied to the multiple alignment indicated that these proteins were rich in α-helix, with no β/extended structure (Figure 1). A phylogenetic tree was constructed based on the amino acid sequences of 55 FAR proteins from 28 species of nematodes (Figure 2). *Ar*-FAR-1 and the FAR-1 protein from *A. besseyi* were in the same branch and had the closest relationship (confidence was up to 99%), and were clustered in the same group with those from other Aphelenchina nematodes. In addition, all these 55 FAR proteins from 28 species of nematodes were divided into six groups: Filarioidea, Ascaridoidea, Tylenchina, Aphelenchina, Strongylida, and Rhabditoidea.

### 2.2. Southern Blot Analysis

**Figure 3:**
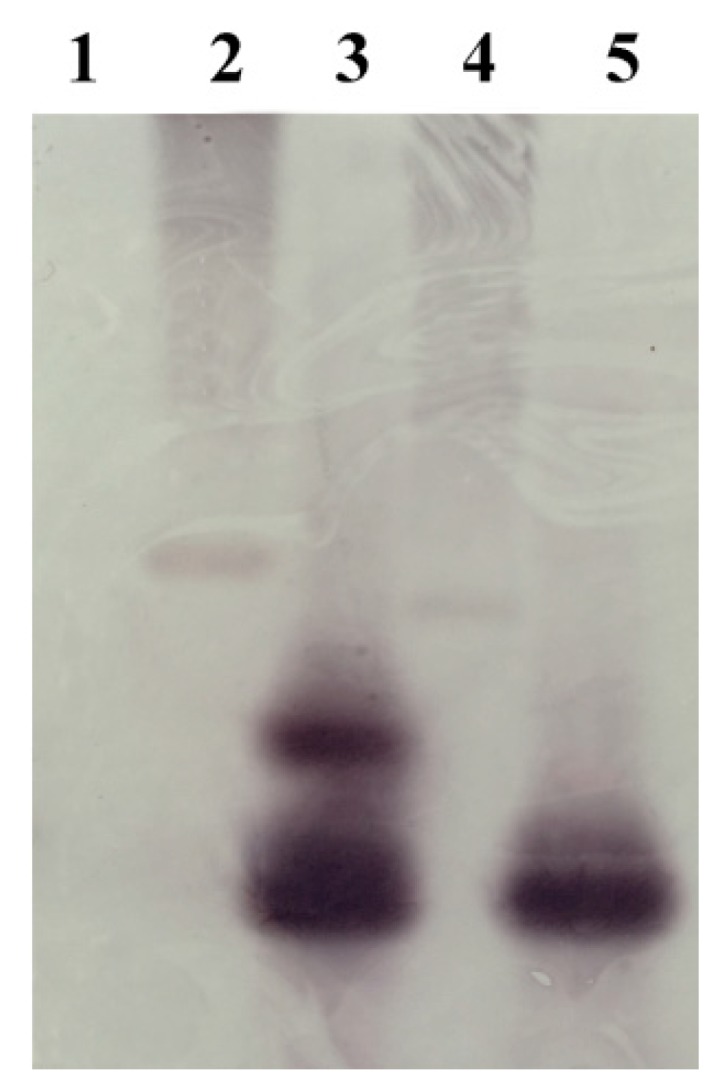
Southern blot analysis of *Ar-far-1* in *Aphelenchoides ritzemabosi.* Lane 1: gDNA from carrot callus digested with restriction enzyme EcoR I; Lane 2: Plasmid pET-32a-*Ar-far-1* digested with restriction enzyme Dra I inside *Ar-far-1* gene; Lane 3: gDNA from A. *ritzemabosi* digested with restriction enzyme Dra I inside *Ar-far-1* gene; Lane 4: Plasmid pET-32a-*Ar-far-1* digested with restriction enzyme EcoR I outside *Ar-far-1* gene; Lane 5: gDNA from A. *ritzemabosi* digested with restriction enzyme EcoR I outside *Ar-far-1* gene.

To determine the gene copy number of the *Ar-far-1* gene in CFN, we analyzed the gDNA of CFN by Southern blot. The results showed that the gDNA digested with restriction enzyme Dra I inside the *Ar-far-1* gene had two signals, and gDNA digested with restriction enzyme EcoR I outside the *Ar-far-1* gene had one signal. No hybridization signal was detected in the digested carrot gDNA (Figure 3). According to the results of southern blot and the positions of restriction enzymes and primers, we determined that the *Ar-far-1* gene had two copies with close positions in gDNA.

### 2.3. Ligand Binding

**Figure 4:**
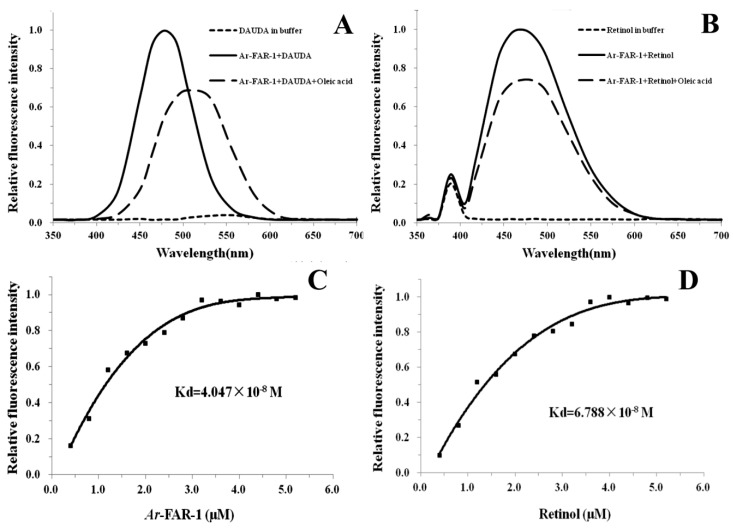
Ligand binding and fluorescent titration lipid binding analysis of r*Ar*-FAR-1. (**A**) Fluorescence emission spectra (excitation at 345 nm) of DAUDA alone or with the addition of r*Ar*-FAR-1. A change in DAUDA emission was observed after the addition of oleic acid to the r*Ar*-FAR-1+DAUDA complex; the peak emission wavelengths for DAUDA were different. (**B**) Fluorescence emission spectra (excitation at 350 nm) of retinol in ethanol alone or after the addition of r*Ar*-FAR-1. The competitive effect of oleic acid was also observed. (**C**) Changes in the relative fluorescence intensity (excitation at 345 nm) of DAUDA (10 mM) in the presence of increasing concentrations of r*Ar*-FAR-1. The curve was used to determine the dissociation constant (K_d_) for the DAUDA/r*Ar*-FAR-1 interaction. (**D**) Change in the relative fluorescence intensity (excitation at 350 nm) of 10 mM r*Ar*-FAR-1 in the presence of increasing concentrations of retinol. The curve was used to derive the equilibrium dissociation constant (K_d_) for the retinol/r*Ar*-FAR-1 interaction.

SDS–PAGE showed that the recombinant *Ar*-FAR-1 protein (r*Ar*-FAR-1) was well purified and appeared as a single band of approximately 20 kDa, which was consistent with the theoretical molecular weight (20.52 kDa) of *Ar*-FAR-1 (Figure S5). The r*Ar*-FAR-1 protein was found to bind the fluorophore-tagged fatty acid 11-(5-dimethylaminonaphthalene-1-sulfonyl amino) undecanoic acid (DAUDA) because the peak emission of DAUDA shifted from 556 nm to 478 nm upon the addition of r*Ar*-FAR-1. After the addition of oleic acid to DAUDA, the protein complex exhibited a pronounced decrease in fluorescence, and the maximum peak emission shifted to 506 nm, indicating that oleic acid can displace DAUDA from the binding site (Figure 4A). The fluorescence emission of retinol was substantially increased when retinol was added to the r*Ar*-FAR-1 solution, and the maximum peak emission shifted from 389 nm to 467 nm. After the addition of oleic acid to retinol, the protein complex exhibited an obvious decrease in fluorescence, and the maximum peak emission shifted to 474 nm (Figure 4B). The titration curve of fluorescence titration experiments predicted a K_d_ of 4.047 × 10^−8^ M for the interaction of r*Ar*-FAR-1 with DAUDA (Figure 4C). We also demonstrated a K_d_ of 6.788 × 10^−8^ M for the interaction of r*Ar*-FAR-1 with retinol (Figure 4D). These results showed that *Ar*-FAR-1 is a fatty acid- and retinoid-binding protein.

### 2.4. Localization and Expression of Ar-far-1 mRNA

**Figure 5:**
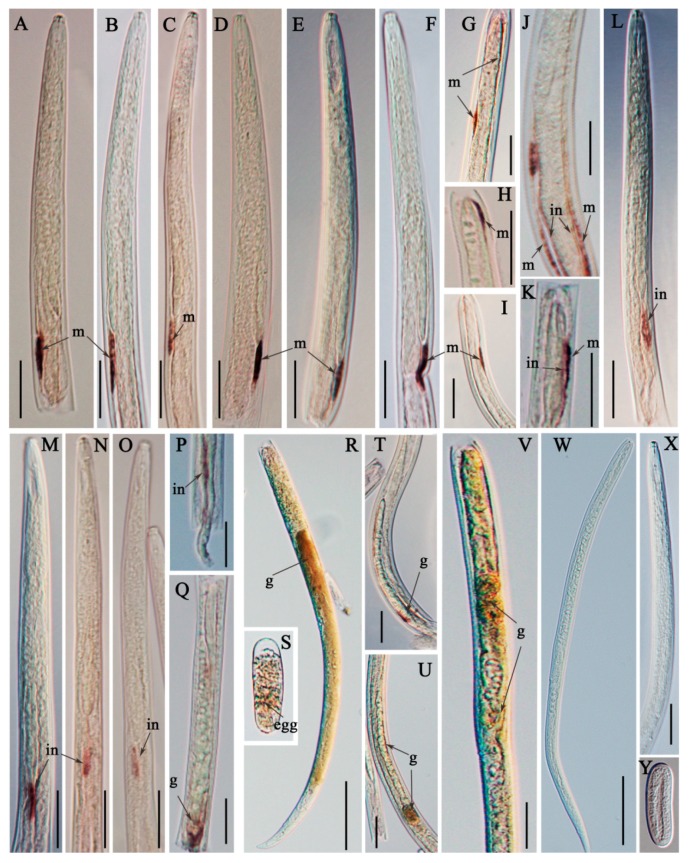
Tissue localization of *Ar-far-1* mRNA in *Aphelenchoides ritzemabosi* using in situ hybridization. *Ab-far-1* mRNA was present in the muscle layers near the end of the esophageal glands (**A**–**F**), as well as in the muscle layers of other parts of the body (**G**–**K**), intestines (**J**–**P**), female genital systems (**Q**–**R** and **T**–**V**) and eggs (**S**). No signal was observed in the nematode sections hybridized with a sense *Ar-far-1* DIG-labeled RNA probe (**W**–**Y**). m: muscle layer; in: intestine; g: female genital system. Scale: A–Q, T–V and X 1 bar = 20 μm; R and W 1bar = 50 μm.

**Figure 6:**
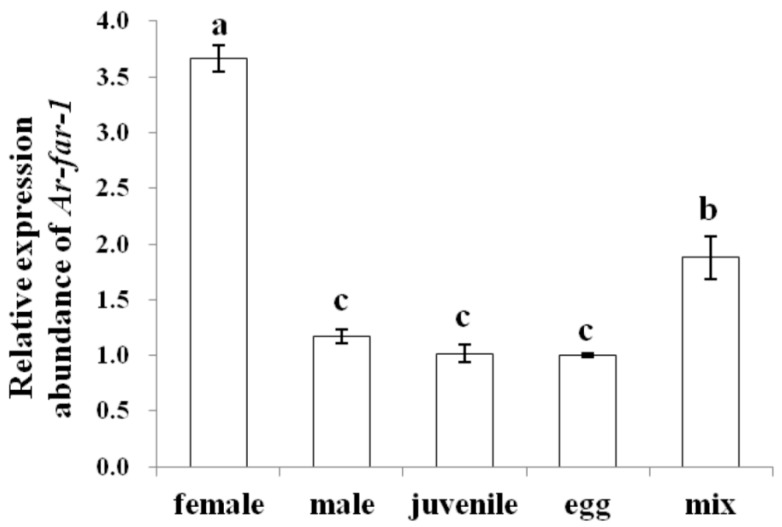
Relative expression of *Ar-far-1* in the mixed-stage nematodes and at different development stages of *Aphelenchoides ritzemabosi.* Mix: mixed-stage nematodes. Bars indicate standard errors of the mean (*n* = 3), and the same letters indicate no significant difference (*p* > 0.05) between treatments according to Duncan’s multiple range test.

The results of in situ hybridization showed that *Ar-far-1* mRNA was mainly localized in the muscle layers (Figure 5A–F) near the end of the esophageal glands, the muscle layers (Figure 5G–K) of other parts of the body, the intestines (Figure 5J–P), and the female genital systems (Figure 5Q–R and 5T–V) and eggs (Figure 5S) of CFN. No hybridization signal was observed in CFN after incubation with the control sense probe (Figure 5J–L). The expression of *Ar-far-1* in females was significantly higher (*p* < 0.05) than that in other developmental stages and mixed-stage nematodes of CFN and was approximately 3.1, 3.6, 3.7, and 2 times higher than that in males, juveniles, eggs, and mixed-stage nematodes, respectively. The expression of *Ar-far-1* was not significantly different (*p* > 0.05) among males, juveniles, and eggs, but *Ar-far-1* expression in these groups was significantly lower (*p* < 0.05) than that in mixed-stage nematodes of CFN (Figure 6).

### 2.5. Ar-far-1 Silencing Efficiency and Influence on the Reproduction and Pathogenicity of CFN

**Figure 7:**
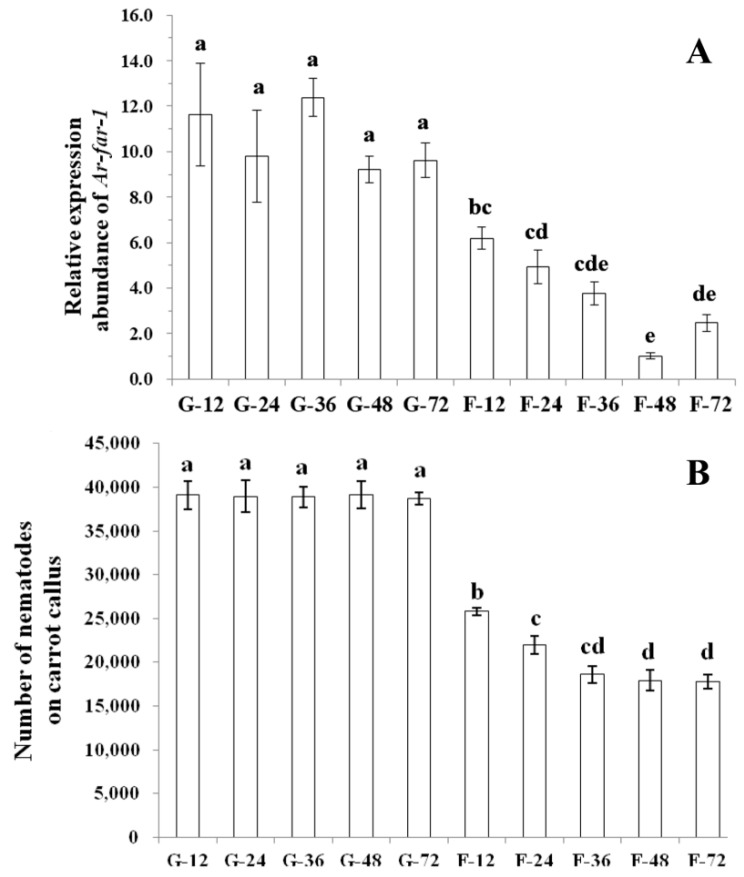
The induction of RNAi in *Aphelenchoides ritzemabosi* by soaking with *Ar-far-1* dsRNA. (**A**) Expression of *Ar-far-1* in *A. ritzemabosi* after different treatments. (**B**) Number of nematodes separated from carrot calluses inoculated with *A. ritzemabosi* under different treatments. G12-G72: *A. ritzemabosi* soaked in *egfp* dsRNA for 12–72 h. F12–F72: *A. ritzemabosi* soaked in *Ar-far-1* dsRNA for 12–72 h. The data are the averages of 3 replicates + standard errors. Bars indicate the standard errors of the means (*n* = 3), and different letters indicate significant differences (*p* < 0.05) between treatments according to Duncan’s multiple range test.

*Ar-far-1* silencing efficiency in CFN was determined by qPCR after nematodes were treated with *Ar-far-1* dsRNA for 12, 24, 36, 48, and 72 h (Figure 7A). The relative expression of *Ar-far-1* in CFN treated with *Ar-far-1* dsRNA decreased significantly (*p* < 0.05) by 46.82%, 49.71%, 69.45%, 88.98%, and 74.25%, respectively, compared with that in the corresponding control CFN treated with *egfp* dsRNA. The relative expression of *Ar-far-1* in CFN treated with *Ar-far-1* dsRNA decreased with the treatment time increased from 12 to 48 h but rebounded at 72 h. The relative expression of *Ar-far-1* in CFN treated with *Ar-far-1* dsRNA was lowest at 48 h, and showed a significant difference (*p* < 0.05) compared with that at 12 and 24 h, but no significant difference (*p* > 0.05) compared with that at 36 and 72 h. There was no significant difference (*p* > 0.05) in the expression of *Ar-far-1* among CFN treated with *egfp* dsRNA.

After being treated with dsRNA for 12, 24, 36, 48, and 72 h and then inoculated onto carrot calluses for 28 days, CFN treated with *Ar-far-1* dsRNA exhibited significantly lower (*p* < 0.05) reproduction than the untreated CFN (CK) and CFN treated with egfp dsRNA at all time points (Figure 7B). Reproduction of the nematodes treated with *Ar-far-1* dsRNA for 36, 48, and 72 h was significantly lower (*p* < 0.05) than that of CFN treated for 12 and 24 h; there was a significant difference (*p* < 0.05) between the reproduction of nematodes treated with *Ar-far-1* dsRNA for 12 and 24 h and no significant difference (*p* > 0.05) between the reproduction of CFN treated with *Ar-far-1* dsRNA for 24 and 36 h (Figure 7B). There was no difference (*p* > 0.05) among the reproduction of nematodes treated with *egfp* dsRNA regardless of the treatment time (Figure 7B).

**Figure 8:**
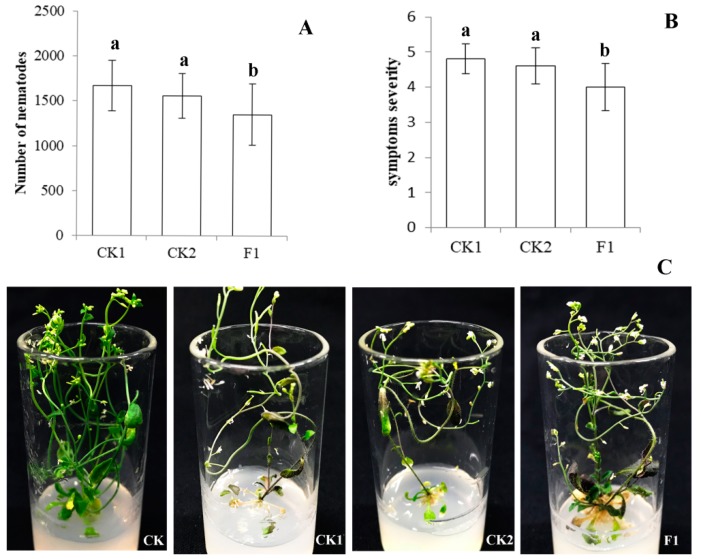
Effect of RNAi on the pathogenicity of *Aphelenchoides ritzemabosi* to *Arabidopsis thaliana. (***A**) Reproduction numbers of *A. ritzemabosi* treated with *Ar-far-1* dsRNA on leaves of the *A. thaliana* Col-0 ecotype. (**B**) Effects of *A. ritzemabosi* on symptom severity in *A. thaliana*. (**C**) Symptoms in *A. thaliana* caused by *A. ritzemabosi*. CK: blank control; CK1, CK2, F1: *A. ritzemabosi* soaked in ddH_2_O, *egfp* dsRNA and *Ar-far-1* dsRNA, respectively, for 48 h. Bars indicate standard errors of the means (*n* = 3), and the same letters indicate no significant difference (*p* < 0.05) between treatments according to Duncan’s multiple range test.

After CFN was treated with ddH_2_O, e*gfp* dsRNA, and *Ar-far-1* dsRNA for 48 h and then used to inoculate the leaves of *A. thaliana* plants for 21 days, the number of nematodes on the *A. thaliana* leaves inoculated with nematodes treated with *Ar-far-1* dsRNA was 1354.87 ± 339.51, which was 81.77% and 86.56% of the number of nematodes on *A. thaliana* leaves inoculated with nematodes treated with ddH_2_O and *egfp* dsRNA, respectively. This difference was significant (*p* < 0.05), but no significant difference (*p* > 0.05) was observed between the latter two treatments (Figure 8A). The severity of the symptoms of *A. thaliana* leaves inoculated with nematodes treated with *Ar-far-1* dsRNA was 4.0 ± 0.67, which was significantly lower (*p* < 0.05) than that of nematodes treated with ddH_2_O and *egfp* dsRNA (Figure 8B,C). Therefore, the pathogenicity of CFN treated with *Ar-far-1* dsRNA was significantly lower than that of the control groups.

## 3. Discussion

The FAR proteins are nematode-specific proteins and play a critical role in many life processes of nematodes. In this study, the full-length cDNA of the FAR-encoding gene *Ar-far-1* of CFN was cloned. The relative expression of *Ar-far-1* in female was the highest among the four developmental stages of CFN, and it was expressed in the muscle layer, intestine, female genital system, and egg. The reproduction and pathogenicity of CFN treated with *Ar-far-1* dsRNA were significantly reduced.

Most of the currently identified FAR proteins from different nematodes were highly conserved [9,10,11,12,13,15,16,22,25]. Like most FAR proteins, *Ar*-FAR-1 has a conserved potential casein kinase II phosphorylation site and protein kinase C phosphorylation site. The phosphorylation of proteins is an important regulatory mechanism that occurs in both prokaryotic and eukaryotic organisms and is critical for many cellular processes [28,29]. In addition, glycosylation is a cotranslational and posttranslational modification that serves a variety of structural and functional roles in the plasmalemma and in secreted proteins [30]. There are differences in the numbers and locations of the potential glycosylation sites in FAR proteins from different species of nematodes and in different FAR proteins from the same species of nematode [8,9,15,21]. However, no potential glycosylation site was found in *Ar*-FAR-1 from CFN in this study. At present, most of the phosphorylation sites and glycosylation sites from FAR proteins have been predicted and found, but whether they are related to the functions of FAR proteins and biological characteristics of the nematodes remains to be further studied.

The expression levels of the *far* genes were different among different species of nematodes and different for *far* genes from the same species of nematode. Similar to the *far-1* genes from *A. besseyi* [15] and *R. similis* [13], *Ar-far-1* from CFN was also the most highly expressed in females, and these three nematode species are migratory plant-parasitic nematodes. The expression levels of *far-1* genes from the sedentary plant-parasitic nematodes, such as *M. hispanica* [23], *M. javanica* [22], and *H. avenae* [12,25], were the highest in juvenile nematodes. The different developmental expression patterns of *far-1* may be related to the biological characteristics of plant-parasitic nematodes. In the mixed stages of migratory plant-parasitic nematodes, the number and biomass of female nematodes are much larger than those of other developmental stages, and females mostly carry out the reproductive and invasive host functions of the species. Thus, females perform more complex physiological functions than other developmental stages. In addition, female development requires more nutrients than other stages. In sedentary plant-parasitic nematodes, the responsibilities of infection and reproduction are mainly borne by juveniles and females, respectively. Therefore, the expression levels of *far* genes may be correlated with the parasitic strategies of plant-parasitic nematodes.

The localization of *far* genes was varied, but most *far* mRNA was detected in the body wall of the nematodes [12,13,15,21,22]. The body wall of a nematode is mainly composed of cuticle, hypodermis, and muscle layer and plays a critical role in regulating the communication between nematodes and the external environment. The location of *far* mRNA in the body wall may help nematodes utilize fatty acids and retinol from the host and the environment to maintain autologous metabolism [13,15]. Of course, more and more *far* mRNAs have been reported to be localized in other parts of the nematodes besides the body wall. For example, *Ace-far-1* mRNA from *Ancylostoma ceylanicum*; and *Ab-far-1*, *Ab-far-2*, and *Ab-far-7* mRNAs from *A. besseyi* were also localized in genital system of nematodes [15,21,27]. *Mhi-far-1* mRNA from *M. hispanica* was localized in the subesophageal gland of second-stage juveniles [23], and *Ha-far-1* mRNA from *H. avenae* and *Ab-far-3* mRNA from *A. besseyi* were localized in intestines of nematodes [12,27]. More interestingly, *Ab-far-3* and *Ab-far-5* mRNAs were also present in the nerve ring of *A. besseyi* [27]. In the study, *Ar-far-1* mRNA was mainly detected in the muscle layer of CFN, especially in the muscle layers near the end of the esophageal glands which were different from the reported *far* mRNA [12,13,15,21,22,25,26,27]. *Ar-far-1* mRNA was also localized in the intestine, female genital system, and egg of CFN. Therefore, FAR proteins may be involved in many biological activities and show diversity in strategies for acquiring fatty acids and retinol in nematodes.

In vitro RNAi played an important role in the validation of gene function in the plant-parasitic nematodes [13,15,25,31,32,33,34,35]. Currently, the function of *far-1* from three species of nematodes has been studied and validated by in vitro RNAi, and these three nematodes were all plant-parasitic nematodes [13,15,25]. The expression levels of *far-1* and the reproductive capacities of the three plant-parasitic nematodes, *R. similis*, *A. besseyi*, and *H. avenae,* were significantly reduced after their *far-1* genes were silenced by in vitro RNAi. The pathogenicity of *R. similis* treated with *Rs-far-1* dsRNA to *Anthurium andraeanum* was significantly reduced, and *Rs-far-1* was shown to be involved in the regulation of AOS expression in *A. thaliana* [13]. The results of this study indicated that the reproduction and pathogenicity of CFN treated with *Ar-far-1* dsRNA on *A. thaliana* were also significantly reduced. These results showed that the *far-1* gene may be involved in the reproduction and infestation of plant-parasitic nematodes. In addition to their function in binding fatty acids and retinols from the environment, FAR proteins may play a role in the transport of fatty acids and retinols, which are used in the development and growth of nematodes [16,17], and also inhibit the plant defense reaction by impeding the metabolism of lipids during plant JA synthesis pathway [10,22]. Therefore, the decrease in reproduction and pathogenicity of the nematodes treated with *far-1* dsRNA may be due to the disturbance of intrinsic metabolism and the weakening of the nematode’s ability to suppress the host immune system, or both. This study was the first to obtain a FAR protein from CFN and validate the characteristic and function of the *far* gene. This could provide a new target to establish an effective method for the control of nematodes based on the nematode-specific *far* genes.

## 4. Materials and Methods

### 4.1. Nematodes

CFN used in this research were collected from the leaves of *Dendranthema morifolium* (Ramat.) Tzvel in Kunming City, Yunnan Province, China. They were maintained by serial subculture on excised carrot (*Daucus carota*) disks in Petri dishes at 25 °C in a dark incubator as previously described [36].

### 4.2. RNA Extraction and Cloning of Ar-far-1 from CFN

Total RNA was extracted using TRIzol reagent (Invitrogen, Carlsbad, CA, USA) and verified as previously described (Cheng et al., 2013). cDNA was synthesized using the RevertAid First Strand cDNA Synthesis Kit (Clontech, Tokyo, Japan), and genomic DNA (gDNA) was extracted with a tissue DNA kit (Magen, Guangzhou, China) according to the manufacturer’s instructions. To study the function of the *Ar-far-1* gene of CFN, the full-length cDNA and gDNA of *Ar-far-1* were amplified using the specific primers, FAR1-F and FAR1-R (Table 1), based on the EST sequence obtained from a previous investigation involving the transcriptome of mixed-stage population of CFN [7]. The PCR products were purified and cloned into the pMD-18T vector (Takara, Tokyo, Japan), transformed into *Escherichia coli* DH5α competent cells and sequenced.

### 4.3. Sequence Analysis, Alignment and Phylogenetics

*Ar-far-1* sequence analysis was performed with Molecular Evolutionary Genetics Analysis version 6.06 software (MEGA 6.06, Tucson, AZ, USA). An ORF was identified and sequence homology comparisons were conducted using the NCBI ORF Finder (http://www.ncbi.nlm.nih.gov/gorf/gorf.html) and the blastx, tblastn and blastp programs from NCBI (http://blast.ncbi.nlm.nih.gov/Blast.cgi), respectively. The signal peptide was predicted with the SignalP 4.1 server [37]. Protein characteristics, including molecular weight, theoretical pI, and protein secondary structure, were predicted using the Protein Machine software available at ExPASy (http://www.expasy.ch/tools/). Multiple protein sequence alignments were conducted with Clustal Omega (http://www.ebi.ac.uk/Tools/msa/clustalo/), and phylogenetic tree analyses were performed with MEGA 6.06 by the neighbor-joining method [38,39].

### 4.4. Southern Blot Hybridization

Primers in Table 1 were designed to amplify the Digoxigenin (DIG)-labeled probe with a PCR DIG Probe Synthesis Kit (Roche, Basel, Switzerland). Approximately 20 μg of gDNA was obtained from CFN and digested with restriction enzymes (Dra I and EcoR I) (Figure S4). The digested DNA products were separated by 0.8% (*w*/*v*) agarose gel electrophoresis and transferred to a Hybond N^+^ membrane (Amersham-Biosciences, Little Chalfont Buckinghamshire, England) [27,40]. The membrane was hybridized for 18 h at 42 °C with the probe. Hybridization was performed using a Dig High Primer DNA Labeling and Detection Starter Kit I (Roche, Basel, Switzerland) according to the manufacturer’s instructions. After hybridization, the membrane was washed with 2 × SSC/0.1% SDS for 15 min at 25 °C followed by 0.5 × SSC/0.1% SDS for 30 min at 65 °C and examined. Equal amounts of carrot callus gDNA and plasmid pET-32a-*Ar-far-1* were used as a control group [39].

### 4.5. Expression and Purification of Recombinant Ar-FAR-1 and Fluorescence-Based Ligand-Binding Assays

To obtain purified *Ar*-FAR-1 protein, full-length *Ar-far-1* was amplified from the plasmid with the primers FAR1FBamHI and FAR1RXhoI (Table 1), and cloned into the prokaryotic expression vector pET-32a (Novagen, Madison, WI, USA). The plasmid was introduced into *E. coli* DH5a for sequence confirmation. Recombinant plasmid DNA was introduced into *E. coli* BL21(DE3) for expression. Expression of the recombinant protein was examined by sodium dodecyl sulfate polyacrylamide gel electrophoresis (SDS-PAGE) and Coomassie brilliant blue staining after treatment with 1 mM isopropyl β-D-Thiogalactopyranoside (IPTG). The recombinant fusion *Ar*-FAR-1 protein with His-tag at the *N*-terminus was purified by affinity chromatography using Ni Sepharose High Performanc (GE Healthcare, Stockholm, Sweden) according to the manufacturer’s instructions. The purity of purified recombinant protein was confirmed by SDS-PAGE [15].

The fatty acid and retinoid binding activity of *Ar*-FAR-1 with signal peptide was detected using the fluorescent analogs DAUDA (Sigma, State College, PA, USA), retinol (Sigma, State College, PA, USA) and oleic acid (Sigma, State College, PA, USA) as previously described [9,10,15]. The dissociation constant (K_d_) values for DAUDA, retinol and oleic acid were estimated as previously described [41].

### 4.6. In Situ Hybridization

Specific sense primers, FAR1-IN-T7F and FAR1-IN-R, and antisense primers, FAR1-IN-F and FAR1-IN-T7R (Table 1), were designed to amplify a 320 bp fragment of *Ar-far-1*. DIG RNA labeling mix (Roche, Basel, Switzerland) was used to synthesize DIG-labeled sense and antisense RNA probes based on the purified PCR product according to the manufacturer’s instructions. The detection of in situ hybridization was performed as described previously [15,39,42].

### 4.7. Expression of Ar-far-1 mRNA at Different Developmental Stages of CFN

To assess the expression levels of *Ar-far-1* at four developmental stages of CFN using qPCR, total RNA samples were extracted from approximately 200 CFN eggs, juveniles, females and males with the RNeasy Micro Kit (Qiagen, Westphalia, Germany) according to the manufacturer’s instructions. The RNA from each sample was used as the template for cDNA synthesis with the ReverTra Ace qPCR RT Master Mix with gDNA Remover (Toyobo, Osaka, Japan) according to the manufacturer’s instructions. The specific primers qPCR-F1 and qPCR-R1 (Table 1); primers Y18s-F and Y18s-R, which are complementary to a reference gene (DQ901554); and primers to amplify 18S rRNA were designed. qPCR was carried out with triplicate technical replicates with iTaq Universal SYBR Green Supermix (Bio-Rad, Hercules, CA, USA) on a CFX96 qPCR instrument (Bio-Rad, Hercules, CA, USA). The initial data analysis was carried out using Bio-Rad CFX96 software as described previously [15,35]. All experiments were performed in three biological replicates with three technical replicates each.

### 4.8. Reproduction and Pathogenicity of CFN after RNAi Treatment

The specific sense primers FAR1-IN-T7F and FAR1-IN-R and the antisense primers FAR1-IN-F and FAR1-IN-T7R (Table 1) were used to amplify *Ar-far-1*. The PCR products were purified, and the sense and antisense single-stranded RNAs (ssRNAs) were transcribed using a Script Max^TM^ Thermo T7 Transcription Kit (Toyobo). The dsRNA was purified as described previously [15,43]. The specific sense primers GFP T7-F and GFP-R and the antisense primers GFP-F and GFPT7-R (Table 1) were designed to amplify a 298 bp fragment from nonendogenous control *egfp* (enhanced green fluorescent protein) dsRNA.

Approximately 500 nematodes separated from excised carrot were collected in DEPC-treated water and then soaked in *Ar-far-1* dsRNA and e*gfp* dsRNA solutions (2.0 mg/mL) for 12 h, 24 h, 36 h, 48 h and 72 h in a dark rotary incubator (100 × *rpm*, 25 °C). The e*gfp* dsRNA solution was used as a negative control. The treated nematodes were washed with DEPC-treated water, total RNA was extracted, and qPCR was used to analyze the transcript levels of *Ar-far-1* mRNA in CFN as described above. Each treatment was replicated three times. In addition, 20 female nematodes soaked in *Ar-far-1* and e*gfp* dsRNA solution (2.0 mg/mL) were then inoculated onto a carrot callus and maintained at 25 °C in a dark incubator for 56 days, after which the nematodes in the carrot callus were isolated, and the total number of nematodes was calculated as previously described [44]. Each treatment was repeated five times. Next, according to Wang’s [2] method, 100 mixed-stage nematodes treated with *Ar-far-1* and e*gfp* dsRNA solution for 48 h were inoculated on *A. thaliana* leaves. The symptoms in *A. thaliana* caused by CFN were tested and the nematodes were extracted from *A. thaliana* after 21 days of inoculation. Each treatment was repeated five times, and each experiment was conducted twice to confirm the results.

### 4.9. Data Analysis

One-way ANOVA was used for all statistical analyses with the SPSS 13.0 statistical software package (SPSS, Inc., Chicago, IL, USA). Similarity between the repeated experiments was tested by preliminary ANOVA using experimental runs as a factor, and no significant differences were detected (*p* > 0.05); therefore, data from two experiments were combined for analyses. Multiple post hoc comparisons were conducted at the 5% level of probability using Duncan’s multiple range rest (DMRT).

## Figures and Tables

**Table 1:** Primers used in this study.

Primer Name	Sequence	Primer Use
FAR1-F	5′-AACCCAAGTTTGAGCAACCTC-3′	cDNA and gDNA amplification
FAR1-R	5′-TATGAAGTTTATTTCGTGTTT-3′	
FAR1-Sb-F	5′-AGAAGTTGTGCCCGAGGAAGT-3′	Southern blot
FAR1-Sb-R	5′-ACCTTCAACAAATGCTTTCGC-3′	
FAR1FBamHI	5′-CGGGATCCATGAGTCTTCGCACATTCGTT-3′	Prokaryotic expression
FAR1RXhoI	5′-CCGCTCGAGTTAATTTTCTTGTTTGATCAA-3′	
FAR1-IN-T7F	5′-GGATCCTAATACGACTCACTATAGGGTACTCTTCCGTTGAGCATTC-3′	*In situ* hybridization and dsRNA template
FAR1-IN-R	5′- GCTTCTCTTCACCCTTAGGT-3′	
FAR1-IN-F	5′- TACTCTTCCGTTGAGCATTC-3′	
FAR1-IN-T7R	5′-GGATCCTAATACGACTCACTATAGGGGCTTCTCTTCACCCTTAGGT-3′	
qFAR1-F	5′-GTCTTCGCACATTCGTTTC-3′	qRT-PCR
qFAR1-R	5′-ATTTTTTCACTTCCTCGGG-3′	
Y18s-F	5′-GACTCAACACGGGAAACCTCA-3′	qRT-PCR
Y18s-R	5′-GCAGACACTCCACACAAGCAC-3′	

The T7 promoter sequence is underlined.

## Data Availability

The transcriptome data from CFN was deposited in NCBI and available under the BioProject accession PRJNA278017, BioSample accession SAMN03401492 and Sequence Read Archive (SRA) accession number SRR3999959. The ORFs and protein sequence of *Ar*-FAR-1 protein was deposited in GenBank under the accession number KX816796.

## Supplementary Materials

Supplementary materials can be found at https://www.mdpi.com/1422-0067/20/22/5566/s1.

## References

[1] Siddiqi M. (1974). Aphelenchoides ritzemabosi. CIH Descriptions of Plant Parasitic Nematodes.

[2] Wang D.W., Peng X.F., Xie H., Xu C.L., Cheng D.Q., Li J.Y., Wu W.J., Wang K. (2016). Arabidopsis thaliana as a suitable model host for research on interactions between plant and foliar nematodes, parasites of plant shoot. Sci. Rep..

[3] Xie H. (2007). The epidemic and detection methods of *Aphelenchoides ritzemabosi*. Plant Quar..

[4] Crozzoli R., Hurtado T., Perichi G., Arcia A. (2008). Characterization of a Venezuelan population of *Aphelenchoides ritzemabosi* on chrysanthemum. Nematol. Mediterr..

[5] Van Megen H., van den Elsen S., Holterman M., Karssen G., Mooyman P., Bongers T., Holovachov O., Bakker J., Helder J. (2009). A phylogenetic tree of nematodes based on about 1200 full-length small subunit ribosomal DNA sequences. Nematology.

[6] Rybarczyk-Mydlowska K., Mooyman P., van Megen N., van den Elsen S., Vervoort M., Veenhuizen P., van Doorn J., Dees R., Karssen G., Bakker J. (2012). Small Subunit Ribosomal DNA-Based Phylogenetic Analysis of Foliar Nematodes (*Aphelenchoides spp*.) and Their Quantitative Detection in Complex DNA Backgrounds. Phytopathology.

[7] Xiang Y., Wang D.W., Li J.Y., Xie H., Xu C.L., Li Y. (2016). Transcriptome Analysis of the Chrysanthemum Foliar Nematode, *Aphelenchoides ritzemabosi* (Aphelenchida: Aphelenchoididae). PLoS ONE.

[8] Garofalo A., Kennedy M.W., Bradley J.E. (2003). The FAR proteins of parasitic nematodes: Their possible involvement in the pathogenesis of infection and the use of *Caenorhabditis elegans* as a model system to evaluate their function. Med. Microbiol. Immun..

[9] Garofalo A., Rowlinson M.C., Amambua N.A., Hughes J.M., Kelly S.M., Price N.C., Cooper A., Watson D.G., Kennedy M.W., Bradley J.E. (2003). The FAR protein family of the nematode *Caenorhabditis elegans*. Differential lipid binding properties, structural characteristics, and developmental regulation. J. Biol. Chem..

[10] Prior A., Jones J.T., Blok V.C., Beauchamp J., McDermott L., Cooper A., Kennedy M.W. (2001). A surface-associated retinol- and fatty acid-binding protein (*Gp*-FAR-1) from the potato cyst nematode *Globodera pallida*: Lipid binding activities, structural analysis and expression pattern. Biochem. J..

[11] Iberkleid I., Sela N., Miyara S.B. (2015). *Meloidogyne javanica* fatty acid- and retinol-binding protein (*Mj*-FAR-1) regulates expression of lipid-, cell wall-, stress- and phenylpropanoid-related genes during nematode infection of tomato. BMC Genom..

[12] Qiao F., Luo L., Peng H., Luo S., Huang W., Cui J. (2016). Characterization of Three Novel Fatty Acid-and Retinoid-Binding Protein Genes (*Ha-far-1*, *Ha-far-2* and *Hf-far-1*) from the Cereal Cyst Nematodes *Heterodera avenae* and *H. filipjevi*. PLoS ONE.

[13] Zhang C., Xie H., Cheng X., Wang D.W., Li Y., Xu C.L., Huang X. (2015). Molecular identification and functional characterization of the fatty acid-and retinoid-binding protein gene *Rs-far-1* in the burrowing nematode *Radopholus similis* (Tylenchida: Pratylenchidae). PLoS ONE.

[14] Basavaraju S.V., Zhan B., Kennedy M.W., Liu Y., Hawdon J., Hotez P.J. (2003). *Ac*-FAR-1, a 20 kDa fatty acid- and retinol-binding protein secreted by adult *Ancylostoma caninum* hookworms: Gene transcription pattern, ligand binding properties and structural characterisation. Mol. Biochem. Parasitol..

[15] Cheng X., Xiang Y., Xie H., Xu C.L., Xie T.F., Zhang C., Li Y. (2013). Molecular Characterization and Functions of Fatty Acid and Retinoid Binding Protein Gene (*Ab-far-1*) in *Aphelenchoides besseyi*. PLoS ONE.

[16] Kennedy M.W., Garside L.H., Goodrick L.E., McDermott L., Brass A., Price N.C., Kelly S.M., Cooper A., Bradley J.E. (1997). The Ov20 protein of the parasitic nematode *Onchocerca volvulus*. A structurally novel class of small helix-rich retinol-binding proteins. J. Biol. Chem..

[17] McDermott L., Cooper A., Kennedy M.W. (1999). Novel classes of fatty acid and retinol binding protein from nematodes. Mol. Cell Biochem..

[18] Garofalo A., Klager S.L., Rowlinson M.C., Nirmalan N., Klion A., Allen J.E., Kennedy M.W., Bradley J.E. (2002). The FAR proteins of filarial nematodes: Secretion, glycosylation and lipid binding characteristics. Mol. Biochem. Parasit..

[19] Solovyova A.S., Meenan N., McDermott L., Garofalo A., Bradley J.E., Kennedy M.W., Byron O. (2003). The polyprotein and FAR lipid binding proteins of nematodes: Shape and monomer/dimer states in ligand-free and bound forms. Eur. Biophys J. Biophy..

[20] Stein L.D., Bao Z.R., Blasiar D., Blumenthal T., Brent M.R., Chen N.S., Chinwalla A., Clarke L., Clee C., Coghlan A. (2003). The genome sequence of *Caenorhabditis briggsae*: A platform for comparative genomics. PLoS Biol..

[21] Fairfax K.C., Vermeire J.J., Harrison L.M., Bungiro R.D., Grant W., Husain S.Z., Cappello M. (2009). Characterisation of a fatty acid and retinol binding protein orthologue from the hookworm *Ancylostoma ceylanicum*. Int. J. Parasitol..

[22] Iberkleid I., Vieira P., Engler J.D., Firester K., Spiegel Y., Horowitz S.B. (2013). Fatty Acid-and Retinol-Binding Protein, *Mj*-FAR-1 Induces Tomato Host Susceptibility to Root-Knot Nematodes. PLoS ONE.

[23] Duarte A., Curtis R., Maleita C., Tiago I., Abrantes I. (2014). Characterization of the venom allergen-like protein (vap-1) and the fatty acid and retinol binding protein (far-1) genes in *Meloidogyne hispanica*. Eur. J. Plant Pathol..

[24] Rey-Burusco M.F., Ibanez-Shimabukuro M., Gabrielsen M., Franchini G.R., Roe A.J., Griffiths K., Zhan B., Cooper A., Kennedy M.W., Corsico B. (2015). Diversity in the structures and ligand-binding sites of nematode fatty acid and retinol-binding proteins revealed by *Na*-FAR-1 from *Necator americanus*. Biochem. J..

[25] Le X.H., Wang X., Guan T.L., Ju Y.L., Li H.M. (2016). Isolation and characterization of a fatty acid- and retinoid-binding protein from the cereal cyst nematode *Heterodera avenae*. Exp. Parasitol..

[26] Phani V., Shivakumara T.N., Davies K.G., Rao U. (2017). *Meloidogyne incognita* Fatty Acid- and Retinol- Binding Protein (*Mi*-FAR-1) Affects Nematode Infection of Plant Roots and the Attachment of *Pasteuria penetrans* Endospores. Front. Microbiol..

[27] Wang D.W., Xu C.L., Ding S.W., Huang X., Cheng X., Zhang C., Chen C., Xie H. (2018). Identification and function of FAR protein family genes from a transcriptome analysis of *Aphelenchoides besseyi*. Bioinformatics.

[28] Cozzone A.J. (1988). Protein phosphorylation in prokaryotes. Annu. Rev. Microbiol..

[29] Stock J.B., Ninfa A.J., Stock A.M. (1989). Protein phosphorylation and regulation of adaptive responses in bacteria. Microbiol. Rev..

[30] Varki A., Etzler M.E., Cummings R.D., Esko J.D. (2009). Discovery and Classification of Glycan-Binding Proteins. Essent. Glycobiol..

[31] Urwin P.E., Lilley C.J., Atkinson H.J. (2002). Ingestion of double-stranded RNA by preparasitic juvenile cyst nematodes leads to RNA interference. Mol. Plant Microbe Interact..

[32] Cottrell T.R., Doering T.L. (2003). Silence of the strands: RNA interference in eukaryotic pathogens. Trends Microbiol..

[33] Chen Q., Rehman S., Smant G., Jones J.T. (2005). Functional analysis of pathogenicity proteins of the potato cyst nematode *Globodera rostochiensis* using RNAi. Mol. Plant Microbe.

[34] Rosso M.N., Dubrana M.P., Cimbolini N., Jaubert S., Abad P. (2005). Application of RNA interference to root-knot nematode genes encoding esophageal gland proteins. Mol. Plant Microbe Interact..

[35] Li Y., Wang K., Xie H., Wang Y.T., Wang D.W., Xu C.L., Huang X., Wang D.S. (2015). A Nematode Calreticulin, *Rs*-CRT, Is a Key Effector in Reproduction and Pathogenicity of *Radopholus similis*. PLoS ONE.

[36] Reise R.W., Huettel R.N., Sayre R.M. (1987). Carrot callus tissue for culture of endoparasitic nematodes. J. Nematol..

[37] Petersen T.N., Brunak S., von Heijne G., Nielsen H. (2011). SignalP 4.0: Discriminating signal peptides from transmembrane regions. Nat. Methods.

[38] Saitou N., Nei M. (1987). The neighbor-joining method: A new method for reconstructing phylogenetic trees. Mol. Biol. Evol..

[39] Wang K., Li Y., Huang X., Wang D.W., Xu C.L., Xie H. (2016). The cathepsin S cysteine proteinase of the burrowing nematode *Radopholus similis* is essential for the reproduction and invasion. Cell Biosci..

[40] Li X.D., Zhuo K., Luo M., Sun L.H., Liao J.L. (2011). Molecular cloning and characterization of a calreticulin cDNA from the pinewood nematode *Bursaphelenchus xylophilus*. Exp. Parasitol..

[41] Cogan U., Kopelman M., Mokady S., Shinitzky M. (1976). Binding affinities of retinol and related compounds to retinol binding proteins. Eur. J. Biochem..

[42] De Boer J.M., Yan Y., Smant G., Davis E.L., Baum T.J. (1998). In-situ Hybridization to Messenger RNA in *Heterodera glycines*. J. Nematol..

[43] Van Rij R.P., Andino R. (2004). RNAi-A guide to gene silencing. Science.

[44] Zhang C., Xie H., Xu C.L., Cheng X., Li K.M., Li Y. (2012). Differential expression of *Rs-eng-1b* in two populations of *Radopholus similis* (*Tylenchida: Pratylecnchidae*) and its relationship to pathogenicity. Eur. J. Plant Pathol..

