# Supplementary material for: Identification and Characterization of a Fatty Acid- and Retinoid-Binding Protein Gene (Ar-far-1) from the Chrysanthemum Foliar Nematode, Aphelenchoides ritzemabosi
Source: Int J Mol Sci. 2019 Nov 7;20(22):5566. doi: 10.3390/ijms20225566 (PMC6888133; doi:10.3390/ijms20225566)
Supplement: Supplementary file 1 [file ijms-20-05566-s001.pdf]

Identification and Characterization of a Fatty Acid- and Retinoid-Binding Protein Gene (*Ar-far-1*) from the Chrysanthemum Foliar Nematode, *Aphelenchoides ritzemabosi*

Shan-Wen Ding, Dong-Wei Wang, Yu Xiang, Chun-Ling Xu and Hui Xie

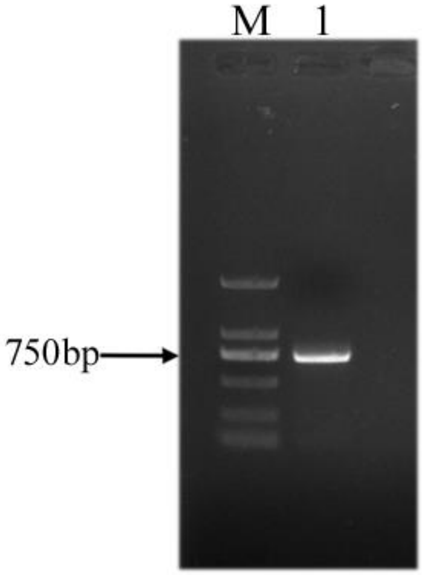

Figure S1. The cDNA sequence amplification results of fatty acid- and retinoid-binding gene (*Ar-far-1*) of *Aphelenchoides ritzemabosi.*

```
Translation of DNAMAN1{RF+1}(31-574)
Universal code
Total amino acid number: 180, MW=20369
Max ORF starts at AA pos 1(may be DNA pos 31) for 180 AA(540 bases), MW=20369

1          AACCCAAGTTTGAGCAACCTCAACAAAACCATGAGTCTTCGCACATTCGTTTCTTTGTTC
1                                          M  S  L  R  T  F  V  S  L  F

61         GCGCTCTTCTGTCTTAGCTATGCAGCTACTCTTCCGTTGAGCATTCATCAAGTGCCCGAA
11          A  L  F  C  L  S  Y  A  A  T  L  P  L  S  I  H  Q  V  P  E

121        CAACTTAAAGAAGTTGTGCCCGAGGAAGTGAAAAAATTCTATGCTGACCTCACTGAAGAA
31          Q  L  K  E  V  V  P  E  E  V  K  K  F  Y  A  D  L  T  E  E

181        GACAAGACCATCTTAAAAGAGGTTGCTGCTAACCACGCCAGCTACGAAAACGAAGATCAA
51          D  K  T  I  L  K  E  V  A  A  N  H  A  S  Y  E  N  E  D  Q

241        GCTATGGAAGCTCTCAAGGCAAAGAGCGAAAAACTGTTTAACAAAGCTACCGAACTTCGT
71          A  M  E  A  L  K  A  K  S  E  K  L  F  N  K  A  T  E  L  R

301        ACTCTCTTGAAAACCAAAATTGACTCGTTGAAACCCGATGCGAAAGCATTTGTTGAAGGT
91          T  L  L  K  T  K  I  D  S  L  K  P  D  A  K  A  F  V  E  G

361        ATCATCAACAAGGTTCGCGCCTTGAAACCTAAGGGTGAAGAGAAGCCTGACTTAAAGAAG
111         I  I  N  K  V  R  A  L  K  P  K  G  E  E  K  P  D  L  K  K

421        ATTCGTGAAGTTGCTAACGAAGTCATCGACTCCTACAAAGCTTTGGGCGAAGAATCTAAA
131         I  R  E  V  A  N  E  V  I  D  S  Y  K  A  L  G  E  E  S  K

481        CAGAACTTGCAAGAAACGTTCCCACAAATCACGAACGTTATTAAGAACGAGAAATTCCAA
151         Q  N  L  Q  E  T  F  P  Q  I  T  N  V  I  K  N  E  K  F  Q

541        ACTCTCGCTCAAGGTTTGATCAAACAAGAAAATTAAACGGTCGTCGAAAACGGATAGCTT
171         T  L  A  Q  G  L  I  K  Q  E  N
```

Figure S2. The amino acid sequence of fatty acid- and retinoid-binding gene (*Ar-far-1*) of *Aphelenchoides ritzemabosi*.

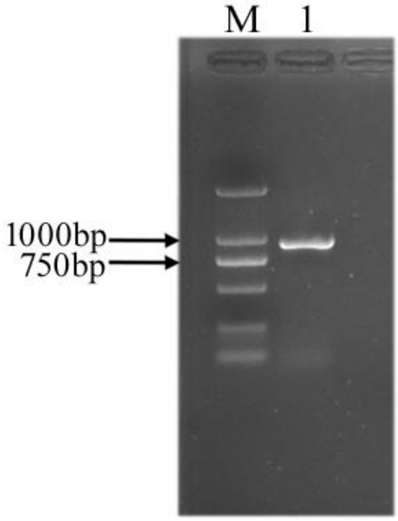

Figure S3. The DNA sequence amplification results of fatty acid- and retinoid-binding gene (*Ar-far-1*) of *Aphelenchoides ritzemabosi.*

```
SEQ  DNAMAN1: 947 bp;
Composition  322 A; 178 C; 169 G; 278 T; 0 OTHER
Percentage:  34.0%  A; 18.8%  C; 17.8%  G; 29.4%  T; 0.0%OTHER
Molecular Weight (kDa): ssDNA: 292.34 dsDNA: 583.70
ORIGIN


1      AACCCAAGTT TGAGCAACCT CAACAAAACC ATGAGTCTTC GCACATTCGT TTCTTTGTTC
61     GCGCTCTTCT GTCTTAGCTA TGCAGCTACT CTTCCGTTGA GCATTCATCA AGTGCCCGAA
121    CAACTTAAAG GTAAAAATCG TGATTACCAT GTTTTTGATT GTTTTAAATT TTAGAAGTTG
181    TGCCCGAGGA AGTGAAAAAA TTCTATGCTG ACCTCACTGA AGAAGACAAG ACCATCTTAA
241    AAGAGGTTGC TGCTAACCAC GCCAGCTACG AAAACGAAGA TCAAGCTATG GAAGCTCTCA
301    AGGCAAAGAG CGAAAAACTG TTTAACAAAG CTACCGAACT TCGTACTCTC TTGAAAACCA
361    AAATTGACTC GTTGAAACCC GATGCGAAAG CATTTGTTGA AGGTGTAAGT TTAAGTCAAT
421    TTTGCACGTA CATAATTTTA AGAATATTAG CTAGCGTGCA AAGCCAATTC AAACTATTAA
481    TTTTCAGATC ATCAACAAGG TTCGCGCCTT GAAACCTAAG GGTGAAGAGA AGCCTGACTT
541    AAAGAAGGTA TTCAATTTTG CATTAAACTT ATTTTATTAA AAGAAACTTT TTTAGATTCG
601    TGAAGTTGCT AACGAAGTCA TCGACTCCTA CAAAGCTTTG GGCGAAGAAT CTAAACAGAA
661    CTTGCAAGAA ACGTTCCCAC AAATCACGAA CGTTATTAAG AGTTAGTTTG CATTCTTATA
721    AAGCTTTCAA ATTTTCGATT TTTTAGACGA GAAATTCCAA ACTCTCGCTC AAGGTTTGAT
781    CAAACAAGAA AATTAAACGG TCGTCGAAAA CGGATAGCTT CGACTAATTA TGTCAACAAT
841    GACCATAGTG ATTTTTGTAA TAATGTGTGT TAAAATTTCG GATCCTTATA GTTGTGCTCG
901    TTTGTTCGAA TAATGTCAGA TCGAAAAAAC ACGAAATAAA CTTCATA
```

Figure S4. The DNA sequence of fatty acid- and retinoid-binding gene (*Ar-far-1*) of *Aphelenchoides ritzemabosi*. The shaded parts were the locations of the four introns. The arrows and box were the positions of the primers of the southern-blot probe and the restriction enzymes Dra I, respectively.

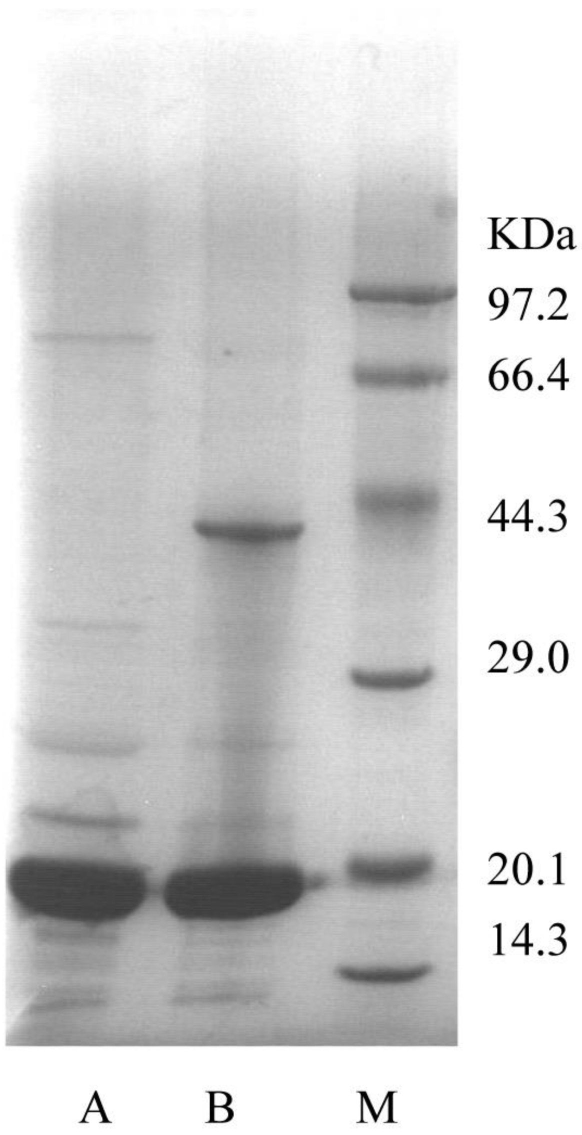

Figure S5. Sodium dodecyl sulphate polyacrylamide gel (SDS-PAGE) of *Escherichia coli* cells expressing fatty acid- and retinoid-binding protein (*Ar*-FAR-1) and its purified protein. M: Protein Marker, A: reduced protein, B: non-reduced protein.

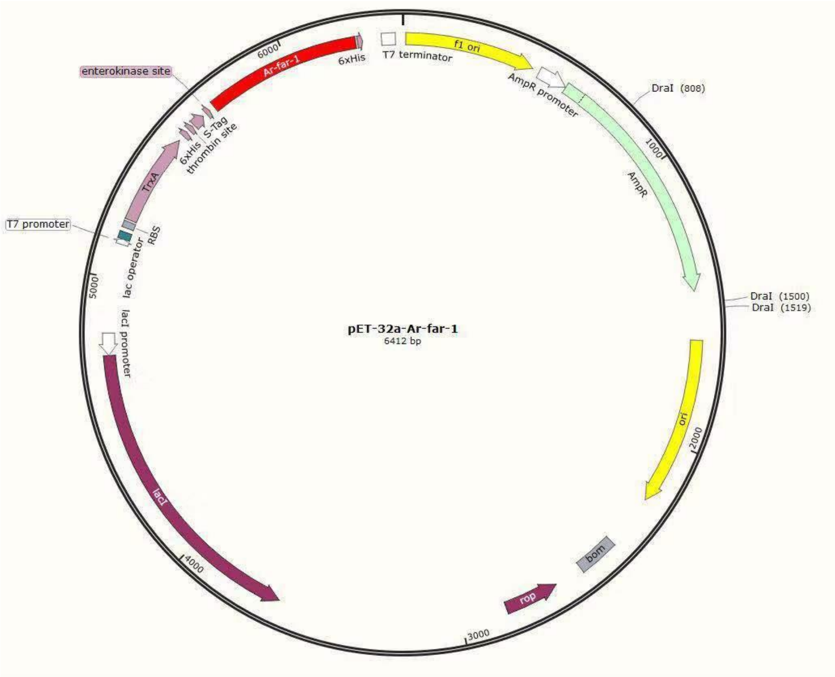

Figure S6. The map of the recombinant vector (pET-32a-*Ar-far-1*). .
